# Two new species of *Cerapanorpa* (Mecoptera, Panorpidae) from the Qinling and Minshan mountains

**DOI:** 10.3897/zookeys.971.55819

**Published:** 2020-09-24

**Authors:** Kai Gao, Meng-Di Li, Bao-Zhen Hua

**Affiliations:** 1 Key Laboratory of Plant Protection Resources and Pest Management, Ministry of Education, Entomological Museum, College of Plant Protection, Northwest A&F University, Yangling, Shaanxi 712100, China Northwest A&F University Yangling China

**Keywords:** biodiversity, China, Panorpidae, scorpionfly, taxonomy

## Abstract

Two new species of *Cerapanorpa* Gao, Ma & Hua, 2016 are described from the Qinling and Minshan mountains, respectively. *Cerapanorpa qinlingensis***sp. nov.** can be readily distinguished from its congeners by the elongate hypovalves and the extremely developed basal process of gonostylus in male genitalia. *Cerapanorpa minshana***sp. nov.** is characterized by its bifurcated parameres and a cluster of long black bristles on the inner apex of the gonocoxite. The number of species of *Cerapanorpa* is raised to 21. An updated key to species of *Cerapanorpa* is presented.

## Introduction

The single-horned scorpionfly *Cerapanorpa* Gao, Ma & Hua, 2016, an endemic genus of Panorpidae (Insecta, Mecoptera) in central China (Gao and Hua 2019), is mainly characterized by a finger-like anal horn on the posterior margin of tergum VI in males, and a medigynium bearing two pairs of basal plates on both sides of the main plate and an elongate rod-like axis with a pair of weakly divergent arms in females (Ma et al. 2012; Gao et al. 2016; Gao and Hua 2019). Species of *Cerapanorpa* are widely distributed in the Qinling-Bashan Mountains and adjacent regions, with an altitude ranging from 1400 m to 2800 m (Gao and Hua 2019). The habitats are cool or humid during their flight period, generally including groundcover in broad-leaf forests, mixed forests, and alpine shrub meadows (Gao and Hua 2019).

The eggs are oval and bear polygonal net-like ridges on the chorion surface (Li et al. 2007; Ma et al. 2009). The saprophagous larvae are eruciform and epedaphic, bearing eight pairs of abdominal prolegs, and usually overwinter as grown larvae in soil cavities (Jiang and Hua 2015; Jiang et al. 2019). The larvae possess a pair of compound eyes consisting of ⁓30 ommatidia (Chen et al. 2012), which almost have the same cellular components as those of their adults although the tiering scheme is different (Chen and Hua 2016). During mating, the male usually secretes a salivary mass as a nuptial gift to attract the female prior to copulation, and uses its single anal horn to clamp the female’s abdominal segment to maintain copulation (Tong et al. 2018).

*Cerapanorpa* currently consists of 19 species (Gao and Hua 2019), which not only display similar internal anatomy (Hou and Hua 2008; Ma et al. 2011), but also have a strongly supported monophyly by phylogenetic analyses (Ma et al. 2012; Hu et al. 2015; Miao et al. 2017, 2019). The alimentary canals are similar in gross morpholgy (Liu and Hua 2009). The male salivary glands uniformly possess six secretory tubes with similar configuration and size (Ma et al. 2011).

Recently, two undescribed species of *Cerapanorpa* were collected from the Qinling and Minshan mountains, a well-known biodiversity hotspot in the world (Myers et al. 2000; Tang et al. 2006; Hu et al. 2019), and are described as new species herein. The number of species of *Cerapanorpa* is raised to 21.

## Material and methods

**Figure 1.**
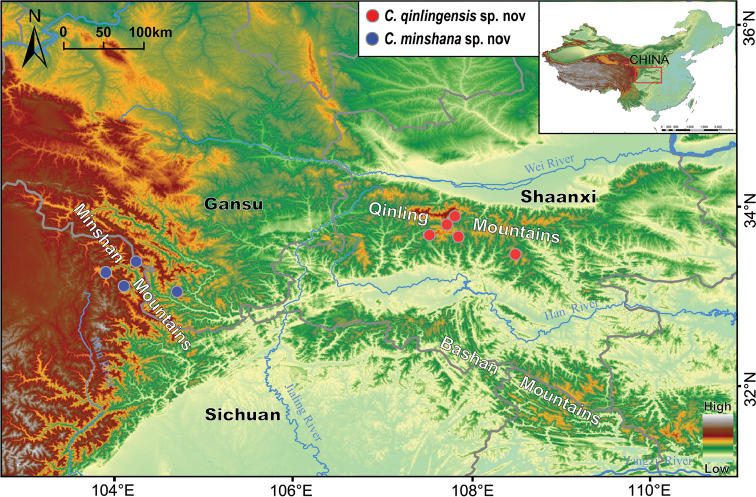
Distribution map of *Cerapanorpa qinlingensis* sp. nov. and *Cerapanorpa minshana* sp. nov.

Specimens were collected from the Qinling and Minshan mountains in central China (Fig. 1), and deposited in the Entomological Museum, Northwest A&F University, China (**NWAU**). Specimens were dissected under a Nikon SMZ 1500 Stereoscopic Zoom microscope. Genitalia were macerated in cold 5% NaOH solution for 3 min and rinsed with distilled water. Wings were measured with a vernier calliper. Adult photographs were taken with a Nikon D7100 digital camera, other images were taken using a scientific digital micrography system, ZEISS SteREO Discovery.V20 equipped with an auto-montage imaging system AxioCam IC. The distribution map was constructed using ArcGIS v10.2 and Adobe Illustrator CC. All photographs were assembled with Adobe Photoshop CS6.

Terminology follows Gao et al. (2016), Hua et al. (2018) and Wang et al. (2019). The following abbreviations and acronyms are applied: A1, first abdominal segment (and so forth for other segments); T1, first tergum (and so forth for other segments).

## Taxonomy

### Cerapanorpa qinlingensis sp. nov.

Taxon classificationAnimaliaMecopteraPanorpidae

AA4E7888-BDA2-5057-B4B6-4A8D3F0FF98F

http://zoobank.org/F0A312C3-EB10-4588-B3AF-326B876FA9E0

Figs 2 , 3 , 4

#### Type material.

***Holotype***: ♂, China: Shaanxi Province, Taibaishan Nature Reserve (33°53'N, 107°48'E), 2100 m, 15 August 2016, leg. Ji-Shen Wang. ***Paratypes***: 2♂5♀, same data as for holotype; 6♂10♀, Zhouzhi County, Qinlingliang (33°49'N, 107°45'E), 2050 m, 24 July 2019, leg. Kai Gao; 12♂18♀, Foping Nature Reserve (33°41'N, 107°52'E), 2200 m, 26 July 2019, leg. Kai Gao; 5♂4♀, Ningshan County, Pingheliang (33°28'N, 108°29'E), 2200 m, 5 July 2019, leg. Xin Tong and Peng-Yang Wang; 3♂, Yangxian County, Changqing Nature Reserve (33°42'N, 107°32'E), 2400 m, 18 July 2019, leg. Yu-Chen Zheng.

#### Etymology.

The specific epithet refers to the type locality, Qinling Mountains.

#### Diagnosis.

The new species resembles *C. emarginata* (Cheng, 1949) in appearance, but can be readily distinguished from the latter by the following characters: 1) wing markings greatly reduced with a faint pterostigmal band (cf. with conspicuous pterostigmal band and apical band); 2) hypovalve longer, reaching the apex of the gonocoxite (cf. shorter, not reaching apex of gonocoxite); 3) paramere shorter, reaching the middle of the gonostylus (cf. longer, reaching apex of gonostylus); 4) gonostylus with an extremely developed basal process (cf. poorly developed).

#### Description of male

**Figure 2.**
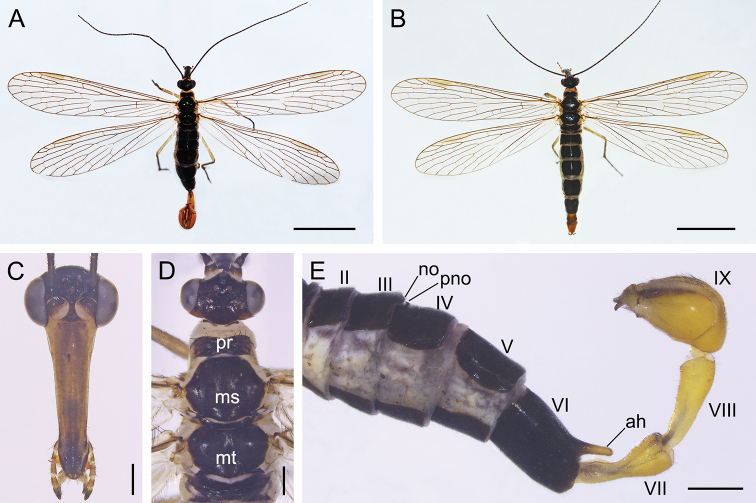
Adults of *Cerapanorpa qinlingensis* sp. nov. **A** male habitus, dorsal view **B** female habitus, dorsal view **C** male head, frontal view **D** male dorsum of head and thorax **E** Male abdomen, lateral view. Abbreviations: **ah** anal horn; **ms** mesonotum; **mt** metanotum; **no** notal organ; **pno** postnotal organ; **pr** pronotum. Scale bars: 5 mm (**A, B**); 0.5 mm (**C–E**).

(Fig. 2A). ***Head*** (Fig. 2C, D). Frons, vertex and occiput brownish black. Rostrum brownish frontally, sparsely covered with short black setae. Maxillary and labial palpi brownish and darkening towards apex. Antennae black and filiform with 38–42 flagellomeres.

***Thorax*** (Fig. 2A, D). Pronotum blackish brown, with 8–12 short setae along its anterior margin. Meso- and metanotum black (Fig. 2D). Pleura light grayish yellow. Legs grayish yellow, with distal tarsomere blackish. Forewing length 13.6–14.4 mm, width 3.4–3.7 mm. Wing membrane hyaline and almost without marking, only with significantly degenerated brown pterostigmal band (Fig. 2A). Hindwing length 12.5–13.6 mm, width 3.2–3.5 mm. Hindwing similar to forewing in markings and patterns.

***Abdomen*** (Figs 2E, 4A). T2–T5 blackish, pleura ivory. Notal organ of T3 very short, not prominent. Postnotal organ of T4 small, hook-shaped and projecting forward. A6 uniformly brownish black, with a brown finger-like anal horn on posterior margin of tergite. A7–A8 elongate and yellowish brown, slightly constricted at base, gradually wider toward apices. A7 with a narrow groove at base.

**Figure 3.**
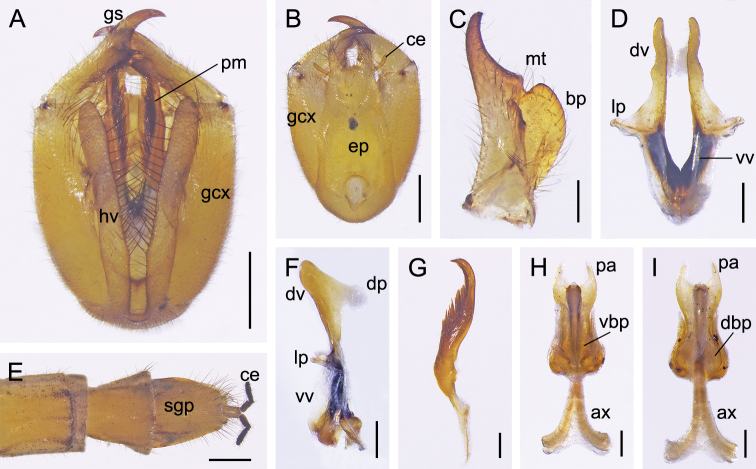
Genitalia of *Cerapanorpa qinlingensis* sp. nov. **A, B** genital bulb, ventral and dorsal views **C** gonostylus, dorsal view **D, F** aedeagus, ventral and lateral views **E** terminalia, ventral view **G** right paramere, ventral view **H, I** medigynium, ventral and dorsal views. Abbreviations: **ax** axis; **bp** basal process; **ce** cercus; **dbp** dorsal basal plate; **dp** dorsal process; **dv** dorsal valve; **ep** epandrium; **gcx** gonocoxite; **gs** gonostylus; **hv** hypovalve; **lp** lateral process; **mp** main plate; **mt** median tooth; **pa** posterior arm; **pm** paramere; **sgp** subgenital plate; **vbp** ventral basal plate; **vv** ventral valve. Scale bars: 0.5 mm (**A, B, E**); 0.2 mm (**C, D, F–I**).

***Male genitalia*** (Fig. 3A, B). Genital bulb yellowish and long oval. Epandrium long and broad, with a nearly trapezoidal emargination distally. Paired hypovalves slender, reaching apex of gonocoxite, bearing a column of long bristles along inner margin. Gonocoxite with a small concave area on apical inner margin, bearing two small protuberances on ventral submedian margin. Gonostylus medially curved, with an indistinct median tooth and an extremely developed basal process on inner margin, and bearing a bundle of short setae dorsally on basal process (Fig. 3C). Parameres extending well beyond base of gonostylus (Fig. 3A), curved distally and pointed apically, bearing a row of dense spines along inner margin (Fig. 3G). Aedeagus sclerotized; dorsal valves of aedeagus long, curved ventrally, with distal part pediform; ventral valves short, membranous; lateral process not prominent (Fig. 3D, F).

#### Description of female.

**Figure 4.**
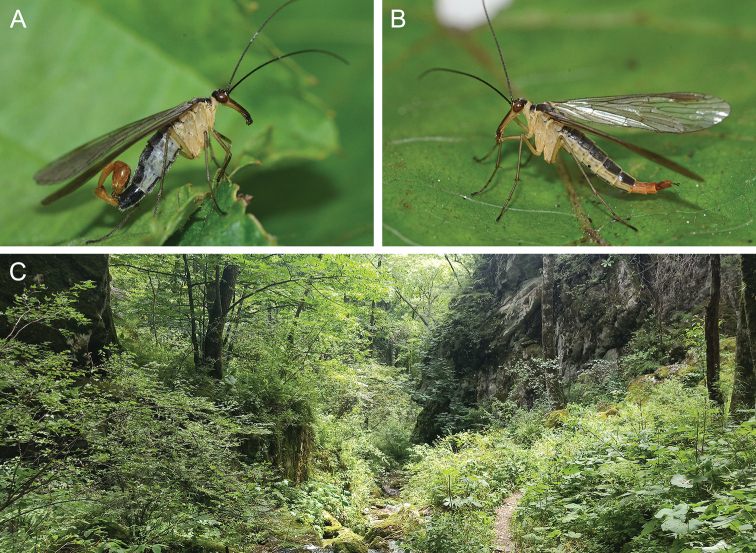
Adult habitus and habitat of *Cerapanorpa qinlingensis* sp. nov. **A** male, lateral view **B** female, lateral view **C** habitat in the southern slope of Mount Taibai.

Similar to the male in wing markings (Figs 2B, 4B). Forewing length 14.3–15.4 mm, width 3.5–3.9 mm; hindwing length 13.8–15.0 mm, width 3.3–3.7 mm, similar to forewings (Fig. 2B).

***Female genitalia*** (Fig. 3E, H, I). Subgenital plate ligulate, not emarginate terminally, bearing long setae on distal portion (Fig. 3E). Medigynium sclerotized, main plate twice as long as wide, intensely constricted medially. Paired posterior arms narrowing apically, forming a broad U-shaped emargination (Fig. 3H, I). Ventral basal plates translucent, covering two-thirds of the main plate (Fig. 3H). Paired dorsal basal plates reniform and membranous (Fig. 3I). Anterior end of axis bifurcated, extending beyond main plate for half its length (Fig. 3H, I).

#### Distribution.

China (Qingling, Shaanxi Province).

#### Habitat.

In the type locality, Taibaishan Nature Reserve, all specimens were captured on the southern slope of the Taibai Mountain, with an elevation of 2100 m. The species mainly inhabits dense herbaceous and shrubby vegetation under evergreen broad-leaved forests (Fig. 4C).

### Cerapanorpa minshana sp. nov.

Taxon classificationAnimaliaMecopteraPanorpidae

6E6A6E31-373E-5A13-819E-50C4118795C0

http://zoobank.org/1E873364-6414-4F8E-8575-053C709D1DD7

Figs 5 , 6 , 7

#### Type material.

***Holotype***: ♂, China: Sichuan Province, Jiuzhaigou County, Anle Town (33°22'N, 104°14'E), 2400 m, 16 June 2019, leg. Kai Gao and Zhi-Chao Jia. ***Paratypes***: 27♂34♀, same data as for holotype; 1♂1♀, Jiuzhaigou County, Majia Town (33°08'N, 104°05'E), 2100 m, 28 May 2019, leg. Kai Gao and Zhi-Chao Jia; 1♀, Jiuzhaigou County, Zhangzha Town (33°16'N, 103°54'E), 2160 m, 19 July 2019, leg. Ning Li and Lu Liu; 18♂22♀, Gansu Province, Wenxian County, Gaoloushan (33°04'N, 104°42'E), 2200 m, 17 June 2019, leg. Kai Gao and Zhi-Chao Jia.

#### Etymology.

The specific epithet refers to the type locality, Minshan Mountains.

#### Diagnosis.

The new species can be readily distinguished from its congeners by the following combination of features: 1) paramere short and bifurcated, bearing a column of long golden spines along the dorsal side; 2) gonocoxite bearing a cluster of black long bristles on the inner apex; 3) dorsal valves of the aedeagus curved ventrally, with the distal part heel-shaped; 4) main plate of medigynium flat, intensely narrowed at the base and broadened distally.

#### Description of male

**Figure 5.**
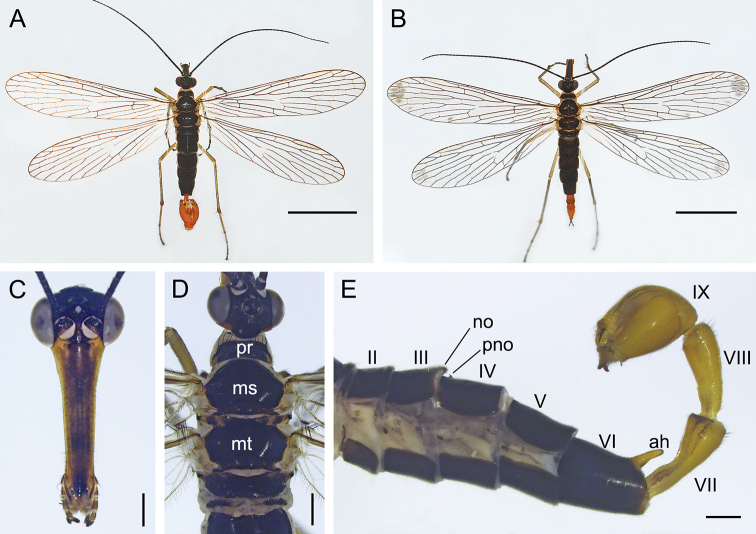
Adults of *Cerapanorpa minshana* sp. nov. **A** male habitus, dorsal view **B** female habitus, dorsal view **C** male head, frontal view **D** male dorsum of head and thorax **E** male abdomen, lateral view. Abbreviations: **ah** anal horn; **ms** mesonotum; **mt** metanotum; **no** notal organ; **pno** postnotal organ; **pr** pronotum. Scale bars: 5 mm (**A, B**); 0.5 mm (**C–E**).

(Fig. 5A). ***Head*.** Frons, vertex and occiput entirely black (Fig. 5C, D). Compound eyes dark gray. Rostrum brownish black anteriorly, mandibles, labial and maxillary palps dark-brown (Fig. 5C). Antennae filiform and black, with 38–43 flagellomeres.

***Thorax*** (Fig. 5D). Pronotum black, with 10–14 black setae along anterior margin. Meso- and metanotum entirely black. Pleura and legs pale yellow with a pair of apical spurs; tarsi darkened toward apices. Forewing length 12.0–12.5 mm, width 3.1–3.3 mm. Wing membrane hyaline, pterostigma and apical band poorly developed, only with dark gray trace at apical region (Figs 5A, 7A). Hindwing length 11.8–12.2 mm, width 2.8–3.2 mm, similar to forewings (Fig. 5A).

***Abdomen*** (Fig. 5A, E). T1–T5 brownish black, pleura pale. Notal organ of T3 semicircular, not prominent. Postnotal organ of T4 small, barb-shaped and projecting forward. T6 brownish black, bearing a yellow finger-like anal horn posteriorly (Fig. 5E). A7 and A8 elongate and uniformly yellowish brown, with basal half slightly constricted and slightly thickened apically.

**Figure 6.**
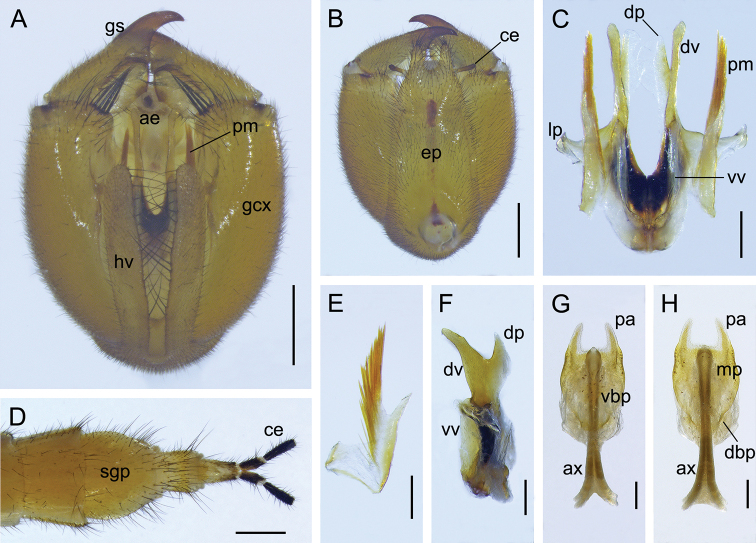
Genitalia of *Cerapanorpa minshana* sp. nov. **A, B** genital bulb, ventral and dorsal views **C** aedeagal complex, ventral view **D** terminalia, ventral view **E** left paramere, lateral view **F** Aedeagus, lateral view **G, H** medigynium, ventral and dorsal views. Abbreviations: **ae** aedeagus; **ax** axis; **ce** cercus; **dbp** dorsal basal plate; **dp** dorsal process; **dv** dorsal valve; **ep** epandrium; **gcx** gonocoxite; **gs** gonostylus; **hv** hypovalve; **lp** lateral process; **mp** main plate; **pa** posterior arm; **pm** paramere; **sgp** subgenital plate; **vbp** ventral basal plate; **vv** ventral valve. Scale bars: 0.5 mm (**A, B**); 0.2 mm (**C–H**).

***Male genitalia*** (Fig. 6A, B). Genital bulb globular and yellowish brown. Epandrium broad basally, narrowing gradually toward apex, with a deep U-shaped emargination between two stout setose lobes (Fig. 6B). Paired hypovalves parallel, only reaching three-quarters of gonocoxite, bearing long bristles along inner margins. Gonocoxite bearing a cluster of black bristles on inner apex (Fig. 6A). Gonostylus shorter than gonocoxite, medially curved, bearing an indistinct middle tooth and a large basal process. Parameres bifurcated and short, not extending beyond the apex of gonocoxite, bearing a column of long golden spines along dorsal side (Fig. 6C, E). Dorsal valves of aedeagus curved ventrally, with distal part heel-shaped (Fig. 6F); ventral valves membranous, weakly developed; lateral process long and curved ventrally.

#### Description of female.

**Figure 7.**
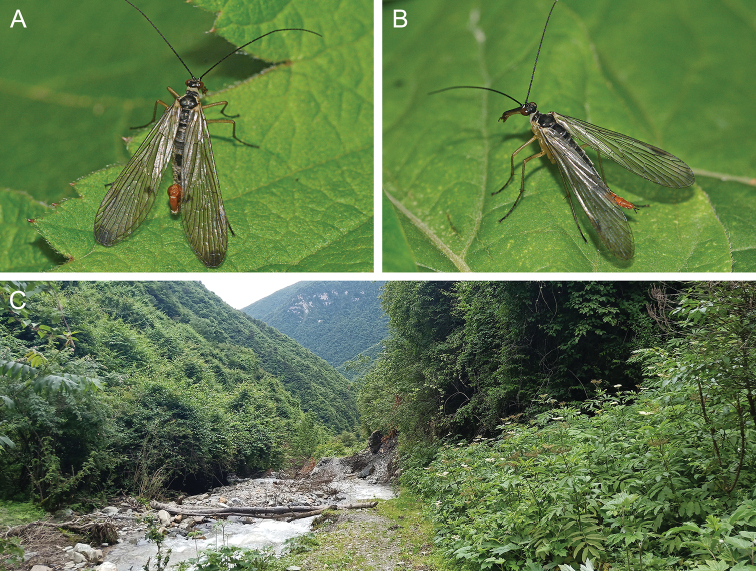
Adult habitus and habitat of *Cerapanorpa minshana* sp. nov. **A** male, dorsal view **B** female, dorso-lateral view **C** habitat of the type locality, Anle Town, Jiuzhaigou County, Sichuan Province, China.

Similar to males in coloration and patterns (Figs 5B, 7B). Forewing length 12.7–13.4 mm, width 3.3–3.7 mm; Hindwing length 12.1–12.5 mm, width 3.1–3.5 mm, similar to forewing (Fig. 5B).

***Female genitalia*.** Subgenital plate long elliptical, ending with a V-shaped incision, bearing long setae on distal portion (Fig. 6D). Medigynium small and weakly sclerotized; main plate flat, intensely narrowed basally, broadened distally (Fig. 6H). Paired posterior arms tapering apically, forming a nearly quadrate emargination. Ventral basal plates membranous and translucent, covering approximately three-quarters of main plate (Fig. 6G). Paired dorsal basal plates oblong, weakly sclerotized (Fig. 6H). Axis elongated and bifurcated anteriorly, extending beyond main plate by nearly half its length (Fig. 6G, H).

#### Distribution.

China (Minshan, Sichuan and Gansu provinces).

#### Habitat.

In the type locality, all specimens were captured on herbaceous groundcover in the Panjiagou Valley (Fig. 7C), with an elevation of 2400 m. Suitable microhabitats in the valley are moist and cool during the imaginal flight period, with the temperature ranging approximately from 14 to 20°C during the day.

### Key to species of *Cerapanorpa* (male)^*^

**Table:** 

1	T5 with an anal horn on posterior margin	***C. bicornifera* (Chou & Wang, 1981)**
–	T5 without anal horns on posterior margin	**2**
2	Finger-like anal horn on T6 shorter and stout, at most 0.2 times as long as T6	***C. brevicornis* (Hua & Li, 2007)**
–	Finger-like anal horn on T6 longer, at least 0.3 times as long as T6	**3**
3	Paramere with thin stalk, then abruptly swollen into broad plate from middle portion	**4**
–	Paramere slightly broader than stalk, with apical portion lanceolate or slightly curved	**7**
4	Paramere bifurcated, with subapical branch	**5**
–	Paramere unbifurcated, without subapical branch	**6**
5	Paramere shorter, only reaching the base of dorsal valves, quadrate plate above the stalk, with an L-shaped subapical branch	***C. byersi* (Hua & Li, 2007)**
–	Paramere longer, reaching apex of dorsal valves, and bearing a column of long golden spines along dorsal side	***C. minshana* sp. nov.**
6	Paramere exceeding apex of gonocoxite, curved medially at apex; dorsal valve not tapering toward apex	***C. baimaensis* Gao & Hua, 2019**
–	Paramere not exceeding apex of gonocoxite, lanceolate at apex; dorsal valve tapering toward apex	***C. centralis* (Tjeder, 1936)**
7	Paramere linear, slightly thicker than stalk, bearing a column of extremely short spines	**8**
–	Paramere flat and broad above stalk, prominently broader than its stalk	**10**
8	Wings with remarkable complete dark-brown markings; middle and hind legs with coxae and trochanters brownish black; paramere curved almost at a right angle basally	***C. reni* (Chou & Wang, 1981)**
–	Wing membrane hyaline, only with faint apical band or without markings; legs with coxae and trochanters yellowish; paramere slightly curved apically	**9**
9	Paramere yellowish brown, blunt apically, reaching middle of gonostylus and bearing spines along inner margin	***C. dubia* (Chou & Wang, 1981)**
–	Paramere dark-brown, reaching apex of gonocoxite and bearing a thorn at apex and spines on dorsal side	***C. liupanshana* Gao, Ma & Hua, 2016**
10	Paramere sinuate or geniculate	**11**
–	Paramere straight and lanceolate, or slightly curved medially	**16**
11	Paramere strongly curved and sinuate in distal half, bearing long comb-like spines along inner margin	**12**
–	Paramere slightly sinuate or geniculate at distal half	**13**
12	Paramere nearly bow-shaped; dorsal valves of aedeagus with truncate apex and membranous apical process	***C. sinuata* Gao, Ma & Hua, 2016**
–	Paramere hook-shaped; dorsal valves of aedeagus tapering toward apex and with large L-shaped apical process	***C. taizishana* Gao & Hua, 2019**
13	Paramere columnar and somewhat sinuate dorsally at apical portion; hypovalves of hypandrium slender and dramatically elongate, exceeding apex of gonocoxite	***C. yanggashana* Gao & Hua, 2019**
–	Paramere geniculate at apical portion; hypovalves of hypandrium exceeding middle of gonocoxite	**14**
14	Rostrum blackish brown to black; paramere with short ventral spines at apex	***C. nanwutaina* (Chou & Wang, 1981)**
–	Rostrum yellowish to reddish brown; paramere with comb-like spines along medial margin	**15**
15	Hypovalves slender, with sparse stout bristles along inner margins; dorsal valves of aedeagus brawny, slightly expanded apically	***C. xuebaodinga* Gao & Hua, 2019**
–	Hypovalves broad, with dense long bristles along inner margins; dorsal valves of aedeagus elongated and slender apically	***C. obtusa* (Cheng, 1949)**
16	Paramere extending to middle of gonostylus	**17**
–	Paramere extending nearly to apex of gonostylus or beyond	**18**
17	Wings only with faint pterostigma and apical bands; paramere with spines from its middle length	***C. funiushana* (Hua & Chou, 1997)**
–	Wings with prominent pterostigmal and apical bands; paramere with a row of short spines on inner margin above basal stalk	***C. wangwushana* (Huang, Hua & Shen, 2004)**
18	Wings without markings; paramere extremely elongated, exceeding apex of gonostylus	***C. protrudens* Gao, Ma & Hua, 2016**
–	Wings with markings; paramere not exceeding apex of gonostylus	**19**
19	Wings only with a faint pterostigmal band; hypovalve elongate, reaching apex of gonocoxite; gonostylus with extremely developed basal process	***C. qinlingensis* sp. nov.**
–	Wings with prominent pterostigmal band and apical band; hypovalve shorter, not reaching apex of gonocoxite; gonostylus with weakly-developed basal process	***C. emarginata* (Cheng, 1949)**

## Discussion

*Cerapanorpa qinlingensis* sp. nov. is endemic to the western Qinling Mountains, and closely related to *C. emarginata* (Cheng, 1949), which is patchily distributed in the eastern Qinling Mountains. The parapatric distribution pattern of these two species probably provides an ideal model to examine the mechanisms of species differentiation or speciation (an east-west genetic break) in the Qinling Mountains, as previously uncovered by phylogeographic studies (Wang et al. 2012, 2013; Liu et al. 2014; Huang et al. 2017).

The discovery of *C. minshana* sp. nov. increases the diversity of the genus *Cerapanorpa* to five species in the Minshan Mountains, including *C. bonis* (Cheng, 1949), *C. baimaensis* Gao & Hua, 2019, *C. xuebaodinga* Gao & Hua, 2019, *C. yanggashana* Gao & Hua, 2019, and *C. minshana* sp. nov. *Cerapanorpa minshana* sp. nov. differs greatly from the aforementioned four species by its bifurcated paramere, the shape of dorsal aedeagal valves and a cluster of black bristles on inner apex of gonocoxite. Only two species, *C. minshana* sp. nov. and *C. centralis* (Tjeder, 1936), possess a cluster of long black bristles on inner apex of gonocoxite in *Cerapanorpa*. However, *C. minshana* sp. nov. can be readily distinguished from *C. centralis* by its specific bifurcated paramere and the shape of dorsal aedeagal valves in males.

*Cerapanorpa qinlingensis* sp. nov. and *C. minshana* sp. nov. are only found in the high-altitude microhabitats of the Qinling and Minshan mountains, respectively (Figs 4C, 7C). These fragmented habitat islands are cool and humid, generally with an altitude above 2000 m (Fig. 1). Most species of *Cerapanorpa* prefer these cool habitats, and usually inhabit the ‘sky islands’ of mountain tops in these mountainous regions (Gao and Hua 2019). Compared with other genera (such as *Panorpa* Linnaeus, 1758 and *Neopanorpa* van der Weele, 1909), which have a broad spectrum of distribution in elevation (Wang and Hua 2017, 2019; Wang et al. 2019), *Cerapanorpa* is likely a cold-adapted genus in Panorpidae.

## Supplementary Material

XML Treatment for Cerapanorpa qinlingensis

XML Treatment for Cerapanorpa minshana

## Footnotes

* Modified from Gao and Hua 2019; male of C. bonis is unknown.
